# Structural Optimization and Performance Analysis of Acoustic Metamaterials with Parallel Unequal Cavities

**DOI:** 10.3390/ma18133087

**Published:** 2025-06-29

**Authors:** Tengyue Pan, Fei Yang, Chengming Jiang, Xinmin Shen, Xiaocui Yang, Wenqiang Peng, Zhidan Sun, Enshuai Wang, Juying Dai, Jingwei Zhu

**Affiliations:** 1 Field Engineering College, Army Engineering University of PLA, Nanjing 210007, China; 13915162583@163.com (T.P.); jcmsweet@163.com (C.J.); sunzhidan0@163.com (Z.S.); dinajy2001@aeu.edu.cn (J.D.); sueecces@163.com (J.Z.); 2 Engineering Training Center, Nanjing Vocational University of Industry Technology, Nanjing 210023, China; 2019101052@niit.edu.cn; 3 MIIT Key Laboratory of Multifunctional Lightweight Materials and Structures (MLMS), Nanjing University of Aeronautics and Astronautics, Nanjing 210016, China; wangenshuai0823@126.com; 4 College of Aerospace Science and Engineering, National University of Defense Technology, Changsha 410073, China; plxhaz@126.com

**Keywords:** acoustic metamaterial, sound-absorbing capacity, theoretical model, acoustic finite element simulation, particle swarm optimization

## Abstract

Noise reduction for manufacturing enterprises is favorable for workers because it relieves occupational diseases and improves productivity. An acoustic metamaterial with parallel, unequal cavities is proposed and optimized, aiming to achieve an optimal broadband sound absorber in the low–frequency range with a limited total thickness. A theoretical model for the acoustic metamaterial of a hexagonal column with 6 triangular cavities and 12 right–angled trapezoidal cavities was established. The lengths of these embedded apertures were optimized using the particle swarm optimization algorithm, with initial parameters obtained from acoustic finite element simulation. Additionally, the impacts of manufacturing errors on different regions were analyzed. The experimental results prove that the proposed acoustic metamaterials can achieve an average absorption coefficient of 0.87 from 384 Hz to 667 Hz with a thickness of 50 mm, 0.83 from 265 Hz to 525 Hz with a thickness of 70 mm, and 0.82 from 156 Hz to 250 Hz with a thickness of 100 mm. The experimental validation demonstrates the accuracy of the finite element model and the effectiveness of the optimization algorithm. This extensible acoustic metamaterial, with excellent sound absorption performance in the low-frequency range, can be mass-produced and widely applied for noise control in industries.

## 1. Introduction

Workers in production and manufacturing industries may suffer pathological damage to their auditory nerves due to long-term exposure to unpleasant noise [1]. The broadband, low-frequency noise within production sites also adversely impacts other bodily functions [2,3,4]. Münzel et al. [2] suggested that transportation noise may be a significant contributor to cardiovascular and cerebrovascular disease. Yu et al. [4] found that the risk of developing cognitive impairment increased with high noise exposure. Therefore, developing an acoustic material that can absorb such noise is of great significance in improving the overall working environment while also protecting employee health.

The low-frequency noise absorption performance of conventional acoustic materials is limited in the restricted space. Artificially designed small-sized acoustic metamaterials with deep subwavelength thickness provide a novel solution to achieve sound absorption at low frequencies. Until now, membrane-type acoustic metamaterials [5,6,7,8,9], Helmholtz resonators [10,11,12,13], and Fabry–Pérot resonators [14,15,16,17] have been presented as effective sound absorbers. Jang et al. [5] developed a deadening material consisting of a filmy membrane combined with an ultra-lightweight membrane-type acoustic metastructure, which exhibited excellent acoustic insulation characteristics with multiple antiresonance frequencies. A novel sound absorber with a Helmholtz resonator, which features a perforated rigid front panel backed by gradually perforated cellular media, was presented by Liu et al. [10]. Long et al. [16] presented an asymmetry-vented sound absorber within a deep subwavelength cavity served by a folded Fabry–Pérot (FFP) resonator and a narrow slit channel. Nevertheless, membrane-type acoustic metamaterials are costly to manufacture and have low strength, making them easily breakable. Additionally, Fabry–Pérot resonators are structurally complex and challenging to fabricate in large quantities. Helmholtz resonators fabricated by light-curing 3D printing are simple, wear- and corrosion-resistant, making them ideal materials for engineering applications.

To achieve a wide range of sound-absorbing performance, a combination or array of multiple acoustic metamaterials is required, as a single structure has a narrow sound absorption frequency range. Bhattacharya et al. [18] presented thin acoustic metastructures based on Ashok Chakra-like metamaterials with subwavelength dimensions, achieving near-perfect sound absorption within the low-frequency domain. Pavan et al. [19] presented a novel cellular maze-like acoustic metastructure that incorporated a folded slit maze-like structure within the microcellular substrate, enabling the substrate to contribute to sound absorption. This structure showed near-perfect sound propagation at low frequencies. Cui et al. [20] investigated an acoustic metamaterial with a coiled Helmholtz chamber comprising three perforated panels, two channeled layers, and a back chamber. The comparison of the sound absorption capacity of this acoustic metastructure with that of the coiled Helmholtz cavity and the common acoustic metamaterial with a normal Helmholtz cavity showed that the presented metastructure achieved lower peak sound-absorbing frequencies. Acoustic metamaterials are mostly composed of square or circular structures [18,19,20]. Circular structural arrays can cause severe space wastage and significantly reduce sound-absorbing capacity in a limited space. Although square structure arrays can achieve excellent absorption performance, the poor mechanical performance causes them to be easily damaged in practical applications. Most existing studies focus on the sound-absorbing capacities of acoustic metamaterials, while the mechanical performance of materials in practical applications is generally overlooked. However, acoustic metamaterials should possess both good sound-absorbing capacity and mechanical performance in practical production sites.

In general, the current challenges for acoustic metamaterials can be summarized as obtaining a higher low-frequency sound absorption performance, a better load-bearing structure, and a smaller occupied space. Therefore, to overcome these problems, an acoustic metamaterial with parallel, unequal cavities that can be arranged in arrays is presented in this study. The aim is to achieve an optimal broad-spectrum sound absorber in the low-frequency range with a limited total thickness by optimizing structural parameters. The hexagonal column is divided into several triangular and trapezoidal columns to achieve wide-range sound absorption in the low-frequency domain, and this honeycomb structure has a better load-bearing capacity. Firstly, a theoretical model [21,22,23] of the acoustic metamaterial with unequal cavities is established to qualitatively analyze the impact of structural parameters on the overall sound-absorbing capacity. Afterward, the initial structural design is completed, and the particle swarm optimization (PSO) algorithm [24,25,26] is used for optimization, with the depth of embedded apertures treated as optimization variables. Additionally, samples have been constructed [27,28,29,30], and the feasibility and accuracy of the designed optimal structure have been verified through experimental testing [31,32,33]. Additionally, the influence of errors on the experimental results is analyzed, providing a useful reference for subsequent engineering applications. The presented acoustic metamaterial and the optimal design methodology provide theoretical guidance for noise reduction.

## 2. Materials and Methods

### 2.1. The Establishment of the Theoretical Model

**Figure 1:**
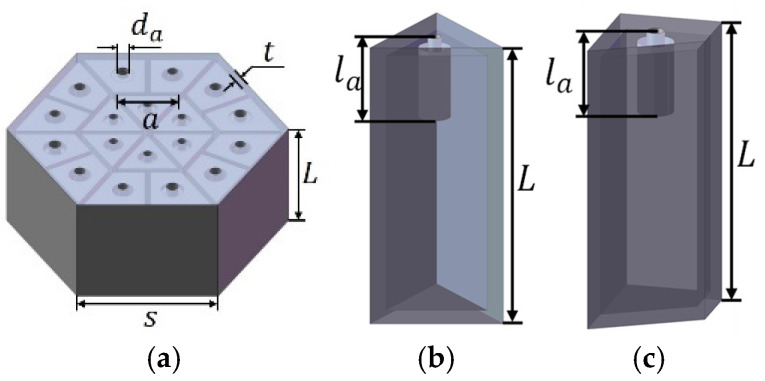
A structural representation of the presented acoustic metamaterial with parallel unequal cavities. (**a**) The overall structure; (**b**) the triangular Helmholtz resonator; and (**c)** the right-angle trapezoidal Helmholtz resonator.

The presented acoustic metamaterial features a hexagonal column divided into 18 basic unit structures, as illustrated in Figure 1a. Each unit structure can be regarded as an independent Helmholtz resonator, with an elongated aperture embedded in the center of each cavity. The triangular and right-angled trapezoidal Helmholtz resonators are presented in Figure 1b and c, respectively. This acoustic metamaterial has a compact structure and good mechanical performance. In Figure 1, the side length and total thickness of the hexagonal column are s and L, respectively; the thickness of each wall is t; the side length of the triangular resonator is a; and for each Helmholtz resonator, the length and diameter of the aperture are l_a_ and d_a,_ respectively.

The sound-absorbing capacity of the acoustic metamaterial is decided by its auditory impedance Z, as shown in Equation (1) [21,22,23].

(1) $$\alpha =1-{\left|\frac{Z-\rho_{0}c_{0}}{Z+\rho_{0}c_{0}}\right|}^{2}$$

Here, $\rho_{0}$ is the density of air under the conditions of room temperature and normal pressure, and $c_{0}$ is the speed of sound in the air at normal temperature and normal pressure [21,22,23].

The auditory impedance Zi of each region can be considered as the sum of two components: $Z_{n}=Z_{a}+Z_{c}$, where $Z_{a}$ is the auditory impedance of the embedded aperture and $Z_{c}$ is the auditory impedance of the internal cavity. Based on the parallel relationship among the different resonators, the overall auditory impedance $Z_{total}$ of the whole acoustic metamaterial is derived, as shown in Equation (2) [21,22,23].

(2) $$Z_{total}=1/\sum_{i=1}^{n}\left(1/Z_{i}\right)$$

When the acoustic waves propagate into a structure, they are attenuated due to thermal and viscous losses. To establish the theoretical model of acoustic wave propagation into the presented acoustic metamaterial [21,22,23], the wave number of viscous waves $k_{v}={\left(-j\omega \rho_{0}/\eta \right)}^{1/2}$ and that of thermal waves $k_{h}={\left(-j\omega \rho_{0}C_{p}/K\right)}^{1/2}$ are introduced, where ω=2πf is the angular frequency, f is the acoustic wave frequency, η is the dynamic viscosity of air, $C_{p}$ is the specific heat at normal pressure, and K is the fluid thermal conductivity. Then, the viscous and thermal fields affecting the acoustic wave propagation are achieved, as shown in Equations (3) and (4), respectively [21,22,23].

(3) $$\psi_{v}=\frac{J_{2}(k_{v}d_{a}/2)}{J_{0}(k_{v}d_{a}/2)}$$

(4) $$\psi_{h}=\frac{J_{2}(k_{h}d_{a}/2)}{J_{0}(k_{h}d_{q}/2)}$$

where $J_{2}$ and $J_{0}$ are the second-order and zero-order Bessel functions of the first type, respectively, and $d_{a}$ is the diameter of the embedded aperture. Then, the complex wave number $k_{c}$, complex air density $\rho_{c}$, and complex sound velocity $C_{c}$ can be calculated using Equations (5), (6), and (7), respectively [21,22,23].

(5) $$k_{c}=k_{0}{\left(\frac{\gamma -(\gamma -1)\psi_{h}}{\psi_{v}}\right)}^{1/2}$$

(6) $$\rho_{c}=\frac{\rho_{0}}{\psi_{v}}$$

(7) $$c_{c}=\frac{\omega }{k_{c}}$$

where γ is the ratio of the specific heat at normal pressure to the specific heat at the normal volume $C_{v}$, i.e., $\gamma =C_{p}/C_{v}$ [21,22,23].

The auditory impedance of a neck-embedded resonator can be expressed as the ratio of the difference in acoustic pressure applied along the diameter of the aperture to the average velocity. Considering the heat loss and the terminal correction resulting from frictional loss of air on the surface of the tube perimeter, the auditory impedance of the neck-embedded triangular cavities and that of the neck-embedded trapezoidal cavities can be expressed by Equations (8) and (9), respectively [21,22,23].

(8) $$Z_{a-tr}=\frac{2\sqrt{3}{\left(a+t\right)}^{2}}{\pi d_{a}^{2}}[-\rho_{0}c_{0}\frac{2j\sin(k_{ca}l_{a}/2)}{\sqrt{(\gamma -(\gamma -1)\psi_{ha})\psi_{va}}}+2\sqrt{2\omega \rho_{0}\eta }]$$

(9) $$Z_{a-trac}=\frac{3\sqrt{3}{\left(a+t\right)}^{2}}{2\pi d_{a}^{2}}[-\rho_{0}c_{0}\frac{2j\sin(k_{ca}l_{a}/2)}{\sqrt{(\gamma -(\gamma -1)\psi_{ha})\psi_{va}}}+2\sqrt{2\omega \rho_{0}\eta }]$$

where a is the side length of the triangle, and $\psi_{va}$, $\psi_{ha}$, and $k_{ca}$ can be calculated by Equations (3)–(5) [21,22,23].

Given the thermal and viscous losses, as well as the terminal correction due to acoustic radiation, the auditory impedance of the triangular and trapezoidal cavities can be expressed as follows [21,22,23].

(10) $$Z_{a-tr}=\frac{2\sqrt{3}{\left(a+t\right)}^{2}}{\pi d_{a}^{2}}\left(-j\frac{S_{a}\rho_{cc}c_{cc}}{\omega V}+j\omega \rho_{0}\delta_{i}\right)$$

(11) $$Z_{a-tra}=\frac{3\sqrt{3}{\left(a+t\right)}^{2}}{2\pi d_{a}^{2}}\left(-j\frac{S_{a}\rho_{cc}c_{cc}}{\omega V}+j\omega \rho_{0}\delta_{i}\right)$$

Owing to the atypical shape of the cavities caused by the embedded aperture, V, it can be expressed as $V=S_{c}L-\pi (d_{a}/2+t)l_{a}$, where $S_{a}$ is the surface area of the aperture and $S_{a}=\pi d_{a}^{2}/4$; $S_{c}$ is the surface area of the cavity, $S_{c-tr}=\sqrt{3}a^{2}/4$, $S_{c-tra}=3\sqrt{3}a^{2}/8$; L is the length of the rear chamber; la is the length of the embedded aperture; and t is the wall thickness. By ignoring the effect of the embedded aperture on the viscous and thermal fields in the cavities, $\rho_{c}$ and $C_{c}$ can be obtained by Equations (6) and (7). $\delta_{i}$ is the terminal correction incurred by acoustic radiation, which can be calculated as follows [21,22,23].

(12) $$\delta_{i}=\left[1+(1-1.25\varepsilon )\right]\times \frac{4}{3}\pi d_{a}$$

For a cylindrical cavity, $\varepsilon =d_{a}/d_{c}$, where $d_{c}$ is the cavity diameter. By approximating the triangular and right-angled trapezoidal cavities as the circular cavities, it can be obtained as $d_{a-triangle}=2\sqrt{S_{c-triangle}/\pi }$ and $d_{a-trapezoid}=2\sqrt{S_{c-trapezoid}/\pi }$ [21,22,23].

Substituting the resulting auditory impedance of the embedded aperture and that of the internal chamber into Equations (1) and (2), the theoretical overall sound-absorbing capacity of this metamaterial can be obtained [21,22,23].

The established theoretical model for the presented acoustic metamaterial with parallel unequal cavities verifies that these structural parameters have a significant impact on the corresponding sound-absorbing capacity. To establish the desired theoretical model, some assumptions and simplifications have been made, which inevitably fail to accurately reflect the actual sound-absorbing capacity of the acoustic metamaterial presented in this study. Thus, adopting optimizations based on the theoretical model, which has been commonly used in previous studies, will lead to significant deviations between the theoretical optimization results and actual measurement results, making it challenging to design a noise reduction product that meets practical requirements. In contrast, the finite element simulation can model the acoustic performance of the metamaterial, referring to a real experimental process, which makes its results closer to the actual measurement results [34]. Hence, optimizations based on the finite element model (FEM) are employed to design acoustic metamaterials featuring parallel, unequal cavities.

### 2.2. Optimization Design

The hardware for data processing is a ThinkStation P3 computing workstation (Lenovo Group, Beijing, China), and the software for acoustic finite element simulation is COMSOL Multiphysics software 5.5 (COMSOL Inc., Stockholm, Sweden). The sound-absorbing capacities of acoustic absorbers primarily depend on intrinsic structural parameters, and a suitable combination of these parameters can achieve a satisfactory sound-absorbing capacity. Thus, adjusting the parameters of the presented acoustic metamaterial to achieve the desired sound-absorbing capacity is a key goal. Considering the optimizations of all structural parameters, achieving wide-range sound absorption in low frequencies is a time-consuming and costly process. Thus, the sound-absorbing capacity in the desired frequency domain is optimized by tuning the length of the embedded aperture using the particle swarm optimization (PSO) algorithm.

In various application scenarios, the desired sound-absorbing capacity of acoustic metamaterials with parallel unequal cavities varies. Here, four different application scenarios are considered to optimize the design of the acoustic metamaterial presented in this study. Based on the on-site measurements, the design requirements are summarized in Table 1. Since the simulated sound-absorbing capacity is typically better than the actual result, a slightly higher performance target is set in structural optimization.

#### 2.2.1. The Design of the Initial Acoustic Metamaterial

**Figure 2:**
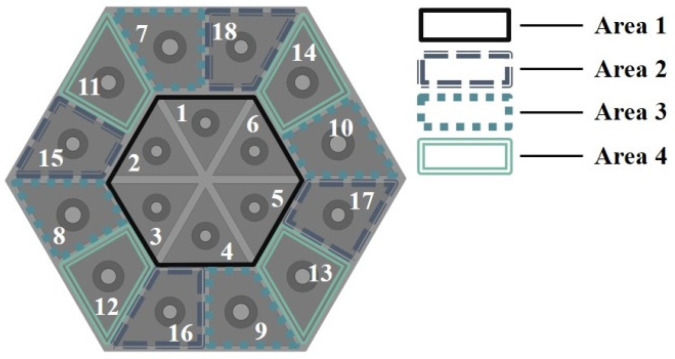
The regional division of the overall structure.

During the optimization process, an excessive number of design variables can lead to an exponential increase in computational load and complexity. To reduce the optimization difficulty, the overall structure is divided into four regions. Herein, the single triangular cavity forms Region 1, and the right-angled single trapezoidal cavity is divided into Regions 2, 3, and 4, with four of them forming a group, as shown in Figure 2.

The design variables are predetermined according to the design requirements. To establish a lightweight structure, the wall thickness is uniformly set at 2 mm. Considering the design requirements of wide-range sound-absorbing capacity in the low-frequency domain, the diameter of the aperture should not be too large or too small. The perforation rates in this study are chosen to range from 0.6% to 0.9%. The diameter of the aperture is obtained from the perforation rate by Equation (13).

(13) $$p=\frac{n\cdot \pi d_{i}^{2}}{3\sqrt{3}s^{2}}$$

where s is the side length of the hexagon and n is the number of single cavities in the region. To achieve an excellent sound-absorbing capacity, the presented structure utilizes the available space effectively, i.e., the thickness of the acoustic metamaterial is equal to the available space. Except for the aperture length, which needs to be optimized, the other parameters of the presented metamaterial are listed in Table 2.

During the structural optimization process, the initial structural parameters randomly assigned by the computer can result in unnecessary calculations, which can consume a significant amount of computational resources and time. Therefore, selecting appropriate initial parameters can shorten the optimization process and improve the efficiency of design optimization. The presented acoustic metamaterial with parallel unequal cavities absorbs sound through the coupling of cavities, with the resonance frequency of the single cavity being around or within the sound-absorbing frequency range of the overall structure. Hence, the computational load can be reduced by tuning the length of the aperture of single cavities so that the resonance frequency is concentrated around the desired frequency domain.

**Figure 3:**
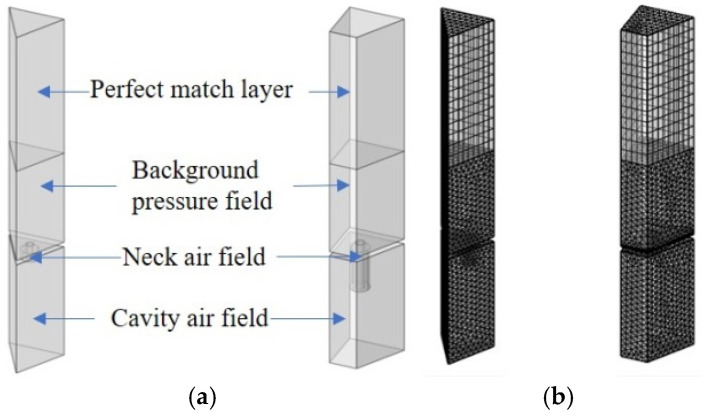
Triangular and right-angled trapezoidal single cavities. (**a**) FEM of the triangular and right-angled trapezoidal single cavities; (**b**) meshed models.

During the design of the initial acoustic metamaterial, the resonance frequencies of single cavities are classified according to the target frequency. In the structural design process, the presented acoustic metamaterial is divided into four regions. Without considering the coupling among the different cavities, the target frequency range is also divided into four frequency ranges, with one region corresponding to one frequency range. The lower the cavity resonance frequency is, the narrower the bandwidth will be. Hence, the division in the low-frequency domain is smaller than that in high-frequency regions. The volume of the triangular cavity is only 2/3 of that of the right-angled trapezoidal cavity, which means that the triangular cavity acts in the low-frequency domain at the expense of certain sound absorption bandwidth, resulting in a narrower overall sound absorption bandwidth. Therefore, to maximize the overall sound absorption bandwidth, the resonance frequencies of the right-angled trapezoidal single cavities 7–14 are in the low-frequency range, while those of the single cavities 1–6 and 15–18 are in the high-frequency range. Thus, the initial resonance frequencies of the 18 single cavities divided according to the 4 application scenarios are shown in Table 3. After the initial resonance frequencies of the 18 single cavities are determined, a FEM is established for the triangular and right-angled trapezoidal single cavities [35,36,37]. As shown in Figure 3a, the model is divided into three parts: the perfect matching layer (PML), the background pressure field (BPF), and the internal air domain. To ensure the accuracy of the finite element simulation, sweeping grids are used for meshing the PML, and free tetrahedral meshes are used to delineate the other regions, as shown in Figure 3b.

In the finite element simulation process, a plane wave acoustic field with an incident pressure amplitude of 1 Pa is generated in the BPF, and the PML represents the infinitely distant air domain, absorbing all outgoing waves. To approach the real measurement process as closely as possible and reduce uncertainty in finite element simulation, extremely fine grids are selected in the grid division.

By constantly reducing the length range of the embedded aperture corresponding to a resonance frequency through parameter scanning, the length of the embedded aperture in the cavity is determined. The obtained parameters of the initial length of the embedded aperture are shown in Table 4. At the same thickness of the acoustic metamaterial for Scenario 1 and Scenario 2, the length of the embedded aperture is longer in Scenario 2 than in Scenario 1 because Scenario 2 has a lower target absorption frequency. Scenario 3 shifts the target frequency range to lower frequencies relative to Scenario 1. The thickness of the acoustic metamaterial for Scenario 3 increases by 20 mm, with little difference in the length of the embedded aperture compared to Scenario 1. The acoustic metamaterial can attenuate the noise influence at lower frequency bands by adjusting both the thickness of the metamaterial and the length of the embedded aperture, which is consistent with the findings of the related study [38,39,40] on Helmholtz resonators and demonstrates the reliability of the presented design method to build the initial structure. When the target frequency is too low, the thickness and length of the embedded aperture are adjusted simultaneously to achieve the target frequency range, as seen in Scenario 4.

**Figure 4:**
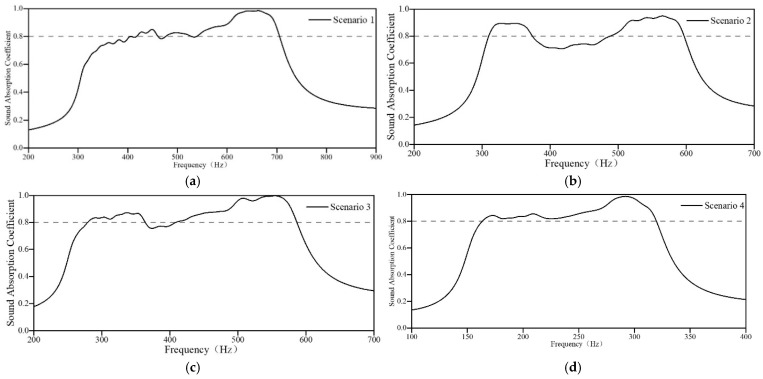
Sound-absorbing capacity of the initial acoustic metamaterial. (**a**) Scenario 1; (**b**) Scenario 2; (**c**) Scenario 3; and (**d**) Scenario 4.

Based on these initial parameters, the sound-absorbing capacities of four initial acoustic metamaterials are obtained, as shown in Figure 4. The four initial acoustic metamaterials resulting from the screening of lengths of individual embedded apertures, according to the target frequency range, all have absorption bands around the target band. The acoustic metamaterial for Scenario 1 exhibits better sound absorption in the high-frequency region than in the low-frequency region, and the low-frequency region does not yet meet the requirements. The acoustic metamaterials for Scenarios 2 and 3 exhibit poor sound absorption in the mid-frequency region. The sound absorption band of the acoustic metamaterial for Scenario 4 leans slightly toward the high-frequency band, but it still does not meet the design requirements. Even if the sound absorption coefficients of non-optimized initial acoustic metamaterials do not yet meet the noise reduction requirements of the application scenarios, the search for optimization has been accelerated considerably.

#### 2.2.2. Structural Optimization by the PSO Algorithm

To enhance the sound-absorbing capacity of structurally complex acoustic metamaterials, optimization algorithms can be employed to solve intricate combinatorial optimization problems. The PSO algorithm, inspired by the foraging behaviors of birds, is simple and easy to implement, and it can effectively solve the design optimization problem of acoustic structures. Hence, the PSO algorithm is employed to optimize the sound-absorbing capacity of the presented acoustic metamaterial.

(1) Establishment of the optimization model: With the total value of the sound absorption coefficient $a\left(f\right)$ in the target frequency domain $\left[f_{\min},f_{\max}\right]$ under the white noise conditions as the objective, the fitness function $f_{fitness}$ is defined as follows:
  (14) $$f_{fitness}=\int_{f_{\min}}^{f_{\max}}\alpha (f)df$$
(2) Determination of decision variable and constraints: optimized frequency range is set at 100–900 Hz; plate thickness t is 2 mm. The length of the embedded aperture of the acoustic metamaterial with parallel, unequal cavities is taken as the decision variable, measured in millimeters (mm).
(3) Determination of the operational parameters and termination conditions of the PSO algorithm: population size N=50; maximum number of iterations N_iterTotal=1000; the weight of self-acceleration $c_{1}=2$; global acceleration weight $c_{2}=2$; and the inertia coefficient $\omega_{0}=0.7$. The iterations end when the current optimal individual is output, either when all absorption coefficients in the optimized frequency range exceed 0.8 or when the maximum number of iterations is reached.

**Figure 5:**
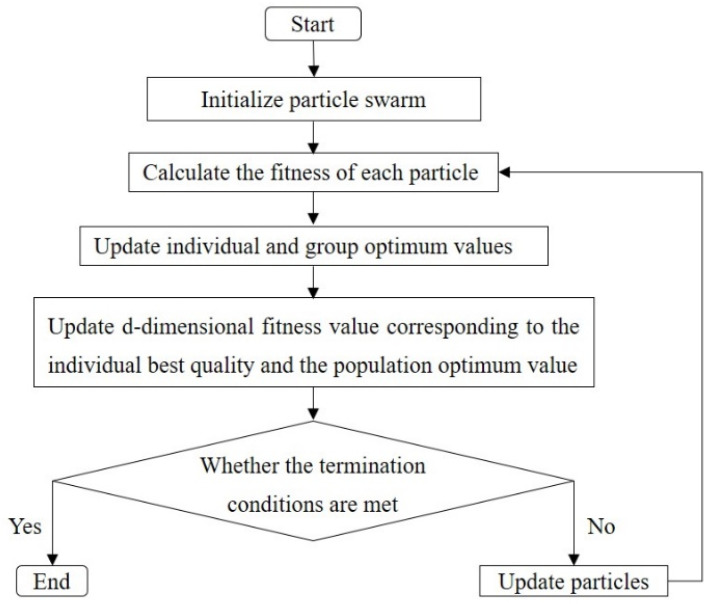
The flow diagram of the PSO algorithm.

The flow diagram of the PSO algorithm used in this study is shown in Figure 5.

The optimized lengths of embedded apertures for four scenarios obtained by the PSO algorithm are shown in Table 5. As the sound-absorbing capacity of the four initial acoustic metamaterials at a high-frequency range significantly exceeds expectations, the lengths of embedded apertures in Region 4 were all increased after optimization, and the length distance of the embedded aperture between the apertures became larger, resulting in a broader frequency band of effect in Region 4, which contributed to improved sound-absorbing capacity in the lower-frequency range. To enable the low- and medium-frequency sound-absorbing capacity in Scenario 1 to meet the design requirements, the length of the embedded aperture in Region 1 increases slightly, and the length of the embedded aperture in Region 2 decreases significantly after optimization. In Scenario 2, there is an obvious reduction in the length of the embedded aperture in Region 1, a slight increase in the length of the embedded aperture in Region 2, and an increase in the length distance of the embedded aperture between apertures, which results in a fuller absorption curve and compensates for the poorer absorption performance in the middle frequency. In Scenario 3, the lengths of all embedded apertures increase, resulting in the absorption performance of the initial acoustic metamaterials shifting toward lower frequencies in the high frequencies. In Scenario 4, the lengths of all embedded apertures in the trapezoidal cavity decrease, and the sound-absorbing capacity shifts toward the target frequency range. This is because the initial acoustic metamaterial operates in the high-frequency range while maintaining sound-absorbing capacity in this region; therefore, the length of the embedded aperture in Region 1 increases.

**Figure 6:**
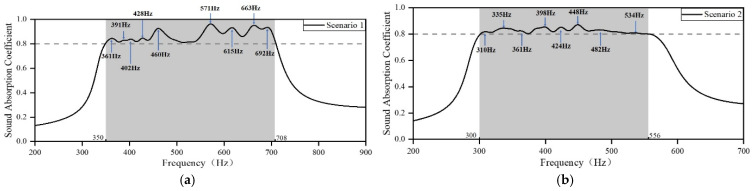

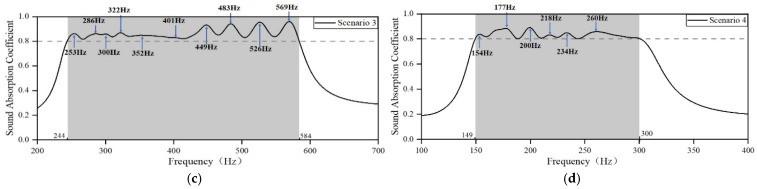
Sound absorption coefficients of the optimized metamaterials. (**a**) Scenario 1; (**b**) Scenario 2; (**c**) Scenario 3; and (**d**) Scenario 4.

By using these parameters from Table 5 in the FEM for simulation analysis, the sound-absorbing curve is obtained, as shown in Figure 6. In this study, the PSO algorithm is employed to design an acoustic metamaterial that achieves high sound absorption capacity in the target frequency region by adjusting the length of the embedded aperture. The presented acoustic metamaterial consists of several resonant elements of different sizes, which facilitate strong absorption of incident acoustic waves with thicknesses less than one order of magnitude of their wavelength, as well as a large absorption bandwidth. It can be observed that four samples with a sound absorption coefficient of 0.8 exhibit sound absorption in the frequency ranges of 350–708 Hz, 300–556 Hz, 244–584 Hz, and 149–300 Hz, which implies that all the structures have good sound-absorbing capacity in a wide frequency range. Scenario 1 includes a total of nine resonance frequency sites, which are 361 Hz, 391 Hz, 402 Hz, 428 Hz, 460 Hz, 571 Hz, 615 Hz, 663 Hz, and 692 Hz; Scenario 2 includes a total of eight resonance frequency sites, which are 310 Hz, 335 Hz, 361 Hz, 398 Hz, 448 Hz, 482 Hz, and 534 Hz; Scenario 3 has a total of ten resonance frequency sites, which are 253 Hz, 286 Hz, 300 Hz, 322 Hz, 352 Hz, 401 Hz, 449 Hz, 483 Hz, 526 Hz and 569 Hz; Scenario 4 has a total of six resonance frequency sites, which are 154 Hz, 177 Hz, 200 Hz, 218 Hz, 234 Hz and 260 Hz. A comparison of acoustic metamaterials for four scenarios reveals that when the structure’s thickness or the length of the embedded aperture increases, the absorption bandwidth shifts to lower frequencies, resulting in improved sound-absorbing capacity in the low-frequency range. Thinner materials with a smaller embedded aperture length occupy less space but inevitably shift the absorption bandwidth to higher frequencies. This suggests that the enhancement in incident acoustic wave absorption and the reduction in device size must be balanced to optimize the material design.

**Figure 7:**
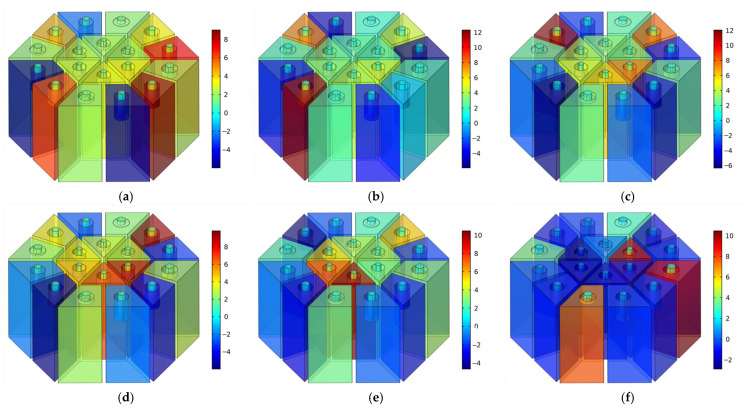

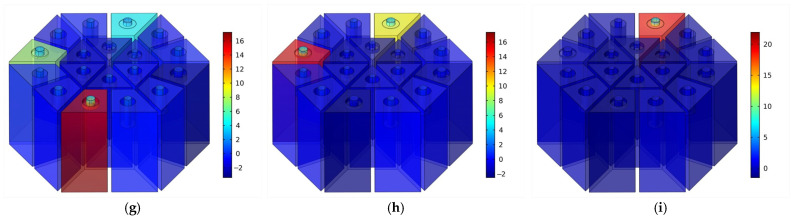
The distribution of sound pressure of optimized acoustic metamaterial for Scenario 1. (**a**) 361 Hz; (**b**) 391 Hz; (**c**) 402 Hz; (**d**) 428 Hz; (**e**) 460 Hz; (**f**) 571 Hz; (**g**) 615 Hz; (**h**) 663 Hz; and (**i**) 692 Hz.

The overall resonance frequency is generated through the coupling of multiple cavities, resulting in a more comprehensive sound absorption curve. To analyze the coupling mechanism for enhancing the sound absorption of the presented acoustic metamaterials with parallel unequal cavities, the optimized acoustic metamaterial for Scenario 1 is considered as an example. The sound pressure distribution diagram at absorption peaks is selected for analysis. The results are shown in Figure 7. The operation mechanism of the presented acoustic metamaterials can be visualized in the diagram as being achieved by the coupling of cavities. When the incident sound pressure wave enters the cavity through the embedded apertures, the air particles flow at a high velocity due to the considerable difference in sound pressure amplitude near the aperture boundary, and the frictional loss leads to sound absorption. At the resonance frequency of 361 Hz, the acoustic waves are concentrated primarily in the cavities of Region 2, resulting in significant energy dissipation. At the resonance frequency points of 391 and 420 Hz, the acoustic waves are mainly concentrated in the cavities of Region 3. At the resonance frequency points of 428 and 460 Hz, the acoustic waves are mainly concentrated in the cavities in Regions 1 and 3. At the resonance frequency point of 571 Hz, the acoustic waves are mainly concentrated in the cavities of Regions 1 and 4. At the resonance frequency points of 615 Hz, 663 Hz, and 692 Hz, the acoustic waves are mainly concentrated in the cavities of Region 4.

## 3. Results and Discussion

### 3.1. Experimental Verification

**Figure 8:**
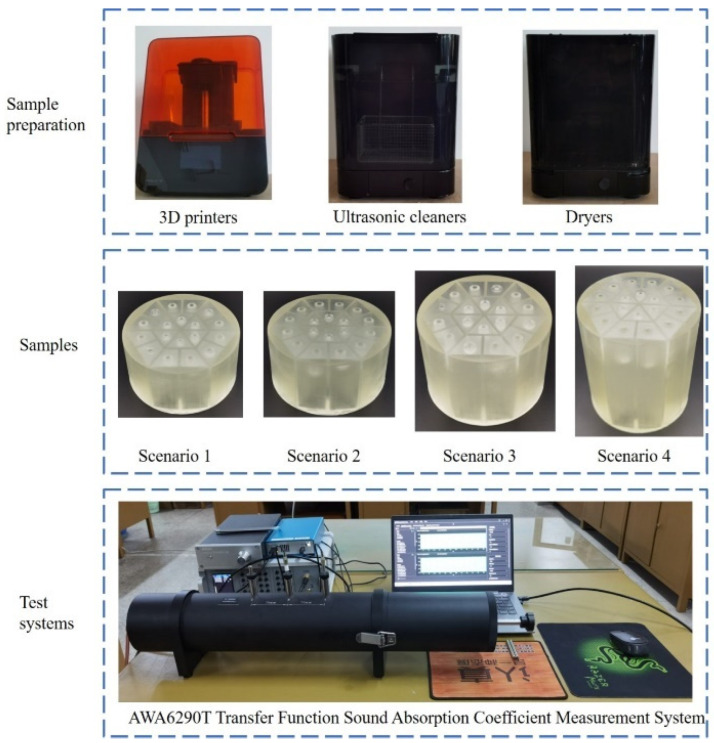
The experimental testing process of acoustic metamaterials.

Sound absorption tests were conducted to validate the reliability of optimization results, as shown in Figure 8. The acoustic samples were manufactured by light-curing 3D printing with a low-force stereolithography 3D printer Form3 (Formlabs Inc., Summerville, MA, USA), cleansed with a Formlabs form wash (Formlabs Inc., Summerville, MA, USA), and dried with a Formlabs Form Cure (Formlabs Inc., Summerville, MA, USA). The samples were prepared as cylindrical samples with the diameter of 100 mm to suit AWA6290T transfer function absorption coefficient measurement system (Hangzhou Aihua Instruments Co., Ltd., Hangzhou, Zhejiang, China) for testing according to the national standard of GB/T 18696.2–2002 (ISO 10534–2:1998) “Acoustics–Determination of sound absorption coefficient and impedance in impedance tubes–part 2: Transfer function method” [31,32,33]. The geometry of the samples did not affect the measurement results. Acoustic absorption of samples was measured by the standing wave method. Figure 8 illustrates the experimental testing process. The prepared sample is installed in the standing wave tube of the AWA6290T system one by one, and the sound absorption coefficients in the concerned frequency ranges can be obtained automatically. For each frequency point, the average value obtained from 200 repeated tests is used as the final result, aiming to minimize random errors and uncertainties in the measurement process as much as possible. The uncertainty in this standing wave measurement process is generally characterized by the variance in multiple measurements, which the equipment manufacturer calibrates to ensure accuracy. The uncertainty of the used AWA6290T system is ±1.5%, which takes into account instrument accuracy, sample sealing performance, fluctuations in environmental conditions, the data processing method, and frequency response characteristics.

**Figure 9:**
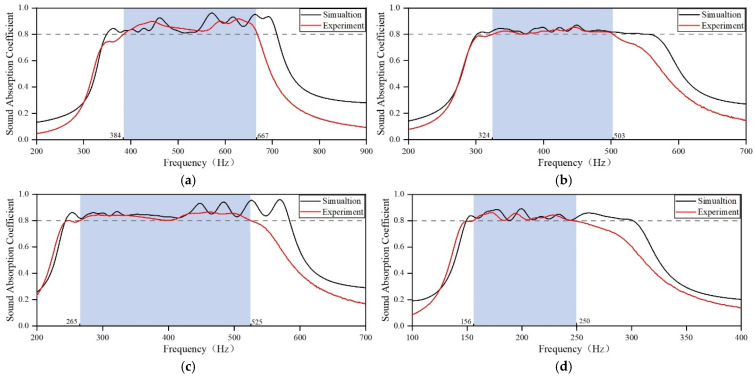
Sound-absorbing curves of the acoustic metamaterial samples. (**a**) Scenario 1; (**b**) Scenario 2; (**c**) Scenario 3; and (**d**) Scenario 4.

The experimental absorption coefficients for the four application scenarios of the samples obtained are shown in Figure 9. The corresponding capacity of the optimized acoustic metamaterial meets the practical application requirements, which validates the accuracy of the presented optimization method. In the actual test, the acoustic metamaterials for Scenarios 1, 2, 3, and 4 can achieve high sound-absorbing capacities in the frequency ranges of 384–667 Hz, 324–503 Hz, 265–525 Hz, and 156–250 Hz, respectively. The experimental results exhibit a fuller sound-absorbing curve due to a tighter coupling among the different cavities.

Due to practical manufacturing errors and the unavoidable gap between the samples and the impedance tube during the testing process, a difference exists between the experimental and simulated results. However, the normal variation trends of the two curves are generally consistent, further verifying the effectiveness of the optimization method. In this study, the acoustic metamaterial is optimized by expanding the bandwidth of the target sound absorption coefficient. Therefore, the designed structure meets the requirements, even if the actual sound-absorbing capacity is slightly worse than the optimized result. During the design process, the same results can also be achieved by optimizing the target sound absorption coefficient.

### 3.2. The Impact of Structural Parameters of Different Regions on the Sound-Absorbing Capacity of the Optimized Structure

According to optimization results, each value is accurate to 2 or even 3 decimal places. However, in the practical manufacturing process, it is not yet possible to achieve such accuracy. With Scenario 1 as an example, the impact of manufacturing errors on the sound-absorbing capacity of the acoustic metamaterial is analyzed, guiding subsequent sample manufacturing and processing. The optimized numerical results are added and subtracted with an equal step size to analyze the impact of structural parameters in different regions on the overall sound-absorbing capacity of the presented acoustic metamaterial.

#### 3.2.1. The Impact of Diameters of Apertures in Different Regions on the Sound-Absorbing Capacity

**Figure 10:**
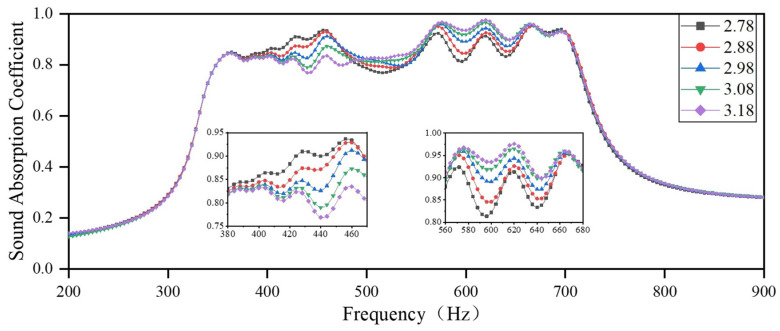
The impact of the diameter of apertures in Region 1 on the sound-absorbing capacity.

The diameters of the apertures in Region 1 are set to 2.69 mm, 2.79 mm, 2.89 mm, 2.99 mm, and 3.09 mm, respectively. The diameters of the apertures in Regions 2, 3, and 4 remain the same as the optimization results. The sound-absorbing curves are shown in Figure 10. As the diameter of the aperture in Region 1 changes, the overall sound absorption coefficient of the acoustic metamaterial also changes. The resonance peak of the triangular cavity shifts to a higher frequency with the increase in the radius of the micro-aperture, resulting in a shift in the overall frequency range in Region 1 to higher frequencies. Indeed, in the low-frequency domain, the material with a small aperture diameter has a better sound-absorbing capacity than that with a large aperture diameter, and the maximum difference in the sound absorption coefficient is 0.13; in the high-frequency domain, the material with a large aperture diameter has better sound-absorbing capacity than that with a small aperture diameter, and the maximum difference in the sound absorption coefficient is 0.12. Meanwhile, the error in the micro-diameter of the aperture increases the fluctuation in the corresponding sound-absorbing curves.

**Figure 11:**
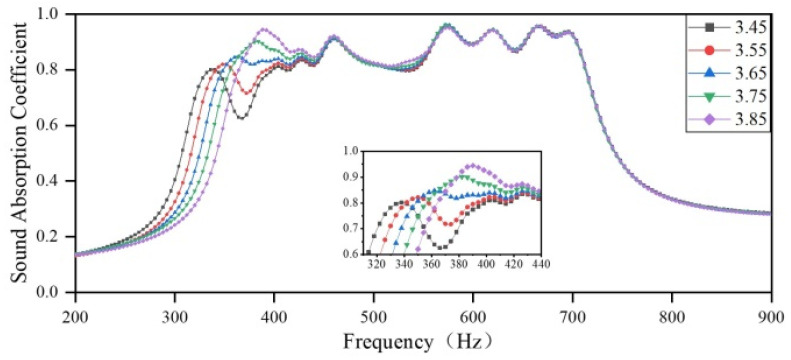
The impact of the diameter of apertures in Region 2 on the sound-absorbing capacity.

Afterward, the influence of variation in the diameter of the aperture in Region 2 on the sound-absorbing curve is explored. The diameter of the aperture in Region 2 is set to 3.33 mm, 3.43 mm, 3.53 mm, 3.63 mm, and 3.73 mm, respectively, and the diameter of the apertures in the other regions remains the same as the optimization results. The sound-absorbing curves are shown in Figure 11. The variation in the diameter of the aperture in Region 2 affects the sound-absorbing capacity in the frequency range of 200–440 Hz. As the diameter of the aperture in Region 2 increases, the resonance peak of the trapezoidal cavity shifts toward higher frequencies, with a maximum shift of 52 Hz. This shift results in the deterioration of the low-frequency sound-absorbing capacity, a decrease in the overall sound absorption bandwidth, and a reduction in the absorption peaks. However, the coupling between Region 2 and Region 3 is enhanced after the shift, resulting in an increased sound absorption coefficient. The maximum difference between the sound absorption coefficients of the valleys between the first and second sound absorption peaks is 0.24. Meanwhile, the sound absorption coefficient in the 400–440 Hz range is improved, while the positions of the peak sound-absorbing frequencies remain unchanged.

**Figure 12:**
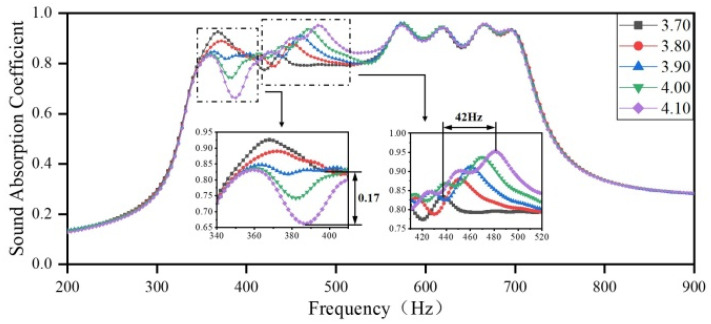
The impact of the diameter of apertures in Region 3 on the sound-absorbing capacity.

Next, the diameter of the aperture in Region 3 is set to 3.70 mm, 3.80 mm, 3.90 mm, 4.00 mm, and 4.10 mm, while the diameters of the apertures in the other three regions remain the same as the optimization results. The sound-absorbing curves are shown in Figure 12. As the diameter of the aperture in Region 3 increases, the resonance peak of the corresponding single cavity shifts toward higher frequency, and the coupling between Region 2 and Region 3 is weakened, while the coupling between Region 1 and Region 3 is enhanced. Furthermore, it is evident that the sound-absorbing capacity in the frequency domain of 340–410 Hz decreases with an increase in the diameter of the aperture, with a maximum difference of 0.17 in the sound absorption coefficients; the absorption peaks also shift slightly toward lower frequencies. In addition, in the high-frequency domain of 410–520 Hz, the sound-absorbing capacity is significantly improved with an increase in the aperture diameter, and the absorption peaks shift toward higher frequencies with an increase in the sound absorption coefficient, with a maximum shift of 42 Hz.

**Figure 13:**
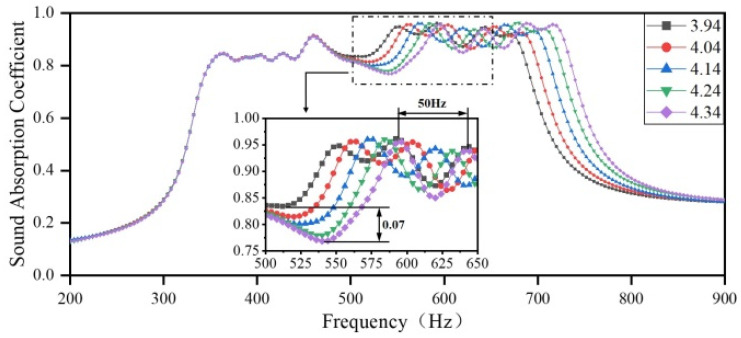
The impact of the diameter of apertures in Region 4 on the sound-absorbing capacity.

Finally, the diameters of the apertures in Region 4 are set to 3.94 mm, 4.04 mm, 4.14 mm, 4.24 mm, and 4.34 mm, respectively. The diameters of the apertures in the other three regions remain the same as those in the optimization results. The sound-absorbing curves are shown in Figure 13. As the diameter of the aperture in Region 4 increases, the sound absorption peaks in the frequency domain, ranging from 500 to 750 Hz, shift toward higher frequencies, with a maximum shift of 50 Hz. The sound absorption coefficient shows a weak variation, with a maximum difference of only 0.07. Furthermore, the coupling between Region 4 and Region 1 is weakened, resulting in a rapid decline of sound-absorbing capacity in the frequency range of 500–570 Hz and a reduced impact of the aperture diameter on the sound absorption coefficient in the high-frequency range. As the diameter of the aperture increases, the sound-absorbing band of the presented acoustic metamaterial becomes wider at the expense of the sound-absorption coefficient.

Overall, it can be concluded that a slight variation in the diameter of the aperture in different regions can significantly impact the overall sound-absorbing capacity. When the diameter of the aperture of a region is changed by only ±0.1 mm and ±0.2 mm, the sound-absorbing ranges of that region, as well as the adjacent regions, change significantly. Furthermore, as the diameter of the aperture increases, the corresponding absorption peaks shift toward higher frequencies and the sound absorption coefficient increases. Therefore, precision machining is required for the diameter of the aperture of the presented acoustic metamaterial in practical machining applications.

#### 3.2.2. The Impact of the Embedded Aperture Length in Various Regions on the Sound Absorption Coefficient

**Figure 14:**
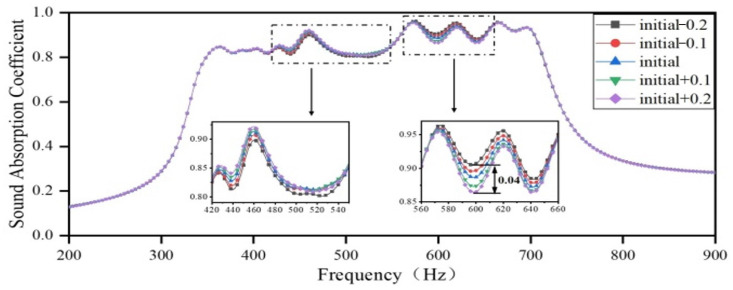
The impact of the length of the embedded aperture in Region 1 on the sound-absorbing capacity.

Firstly, the length of the embedded aperture in Region 1 is increased by 0.1 mm and decreased by 0.2 mm, respectively, while the lengths of the embedded apertures in Regions 2, 3, and 4 remain unchanged, as per the optimization results. The sound-absorbing curves are shown in Figure 14. As the length of the embedded aperture in Region 1 increases, the resonance peak of the cavity in Region 1 shifts toward a lower frequency, and the coupling between Region 2 and Region 4 is weakened, while the coupling between Region 1 and Region 3 is enhanced. Furthermore, as the length of the embedded aperture increases, the sound-absorbing capacity in the frequency domain of 420–560 Hz is slightly enhanced, while that in the frequency domain of 560–660 Hz is slightly deteriorated. Since the sound-absorbing curve in the original frequency domain of Region 1 is relatively flat, the change in the length of the embedded aperture has a negligible impact on absorption peaks in the overall sound-absorbing curve.

**Figure 15:**
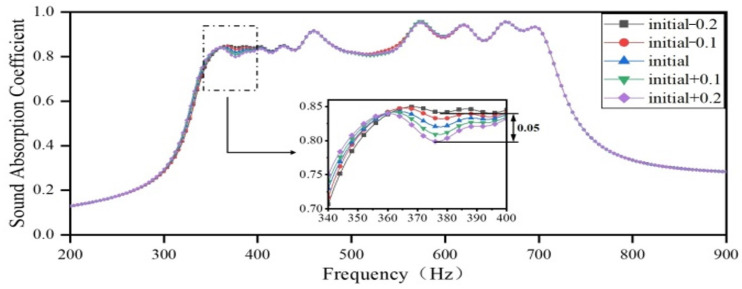
The impact of the length of the embedded aperture in Region 2 on the sound-absorbing capacity.

Next, the lengths of the embedded apertures in Region 2 are increased by 0.1 mm and decreased by 0.2 mm, respectively, while the lengths of the embedded apertures in the other three regions remain unchanged, as per the optimization results. The sound-absorbing curves are shown in Figure 15. As the length of the embedded aperture in Region 2 increases, the coupling between Region 2 and Region 3 is weakened, the absorption peaks in the frequency range 350–370 Hz slightly shift toward lower frequency, the sound absorption coefficient slightly decreases, and the valleys show the most significant decrease, with a maximum difference of only 0.05 in the sound absorption coefficient.

**Figure 16:**
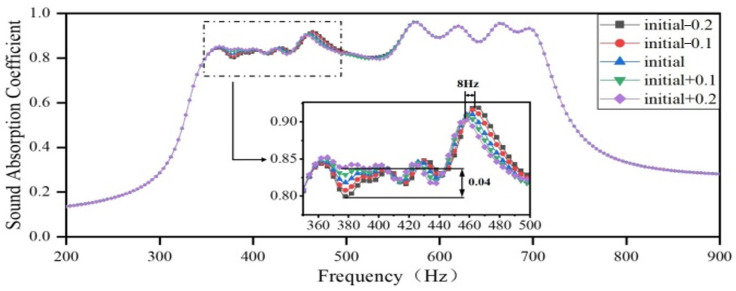
Impact of the length of the embedded aperture in Region 3 on the sound-absorbing capacity.

Then, the lengths of the embedded apertures in Region 3 are increased by 0.1 mm and decreased by 0.2 mm, respectively, and the lengths of the embedded apertures in the remaining three regions remain the same as the optimization results. The sound-absorbing curves are shown in Figure 16. As the length of the embedded aperture in Region 3 increases, the resonance peak of the single cavity in Region 3 slightly shifts toward a lower frequency. Furthermore, the sound-absorbing curve in the frequency domain, between 400 and 500 Hz, slightly shifts to a lower frequency, with a maximum shift of 8 Hz. Additionally, the coupling between Region 3 and Region 2 is strengthened, while that between Region 3 and Region 1 is weakened. Consequently, the sound absorption coefficients in the frequency domain 350–400 Hz slightly increase, with a maximum difference of 0.04, and the sound absorption coefficients in the frequency domain 400–500 Hz slightly decrease.

**Figure 17:**
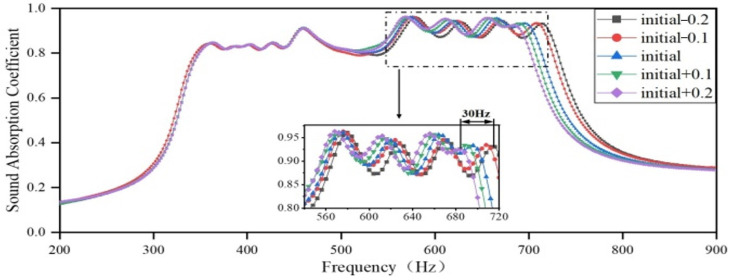
The impact of the length of the embedded aperture in Region 4 on the sound-absorbing capacity.

Finally, the lengths of the embedded apertures in Region 4 are increased by 0.1 mm and decreased by 0.2 mm, respectively, while the lengths of the embedded apertures in the other three regions remain unchanged, as per the optimization results. The sound-absorbing curves are shown in Figure 17. Since the length of the original embedded aperture in Region 4 is small, changing the length of the aperture in Region 4 by ±0.1 and ±0.2 mm indicates a change of ±4.5% and ± 9% concerning the original value. Hence, a slight variation in the length of the embedded aperture in Region 4 can significantly impact the sound-absorbing curve. An increase in the length of the embedded aperture in Region 4 can cause a shift in the absorption peaks toward a lower frequency, with a maximum shift of 30 Hz, a decline in the sound-absorbing capacity in the high-frequency domain, a slight reduction in the sound absorption bandwidth, and a slight improvement in the sound absorption coefficient for a low-frequency domain.

Overall, it can be concluded that when the cavity has an original embedded aperture with a large length, such as in Regions 1, 2, and 3, the variation in the length of the embedded aperture has a minor influence on the overall sound-absorbing capacity. However, when the cavity has an original embedded aperture with a small length, such as in Region 4, a minor error in the aperture length can significantly impact the overall sound-absorbing capacity. Therefore, precision machining is required when machining materials with a small embedded aperture length to ensure an excellent sound-absorbing capacity.

#### 3.2.3. The Impact of the Depth of the Rear Cavity on the Sound Absorption Coefficient

**Figure 18:**
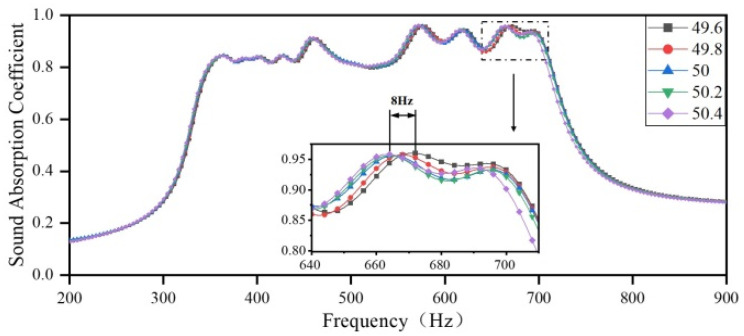
The impact of the depth of the rear cavity on the sound-absorbing capacity.

Here, the influence of a manufacturing error in the depth of the rear cavity on the sound-absorbing capacity is examined. The depths of the back cavities are set to 49.6 mm, 49.8 mm, 50 mm, 50.2 mm, and 50.4 mm, respectively. The obtained sound-absorbing curves are shown in Figure 18. As the depth of the rear cavity changes, the sound-absorbing curve of the presented acoustic metamaterial changes by 0.4% and 0.8% compared to that with the depth of the original rear cavity. The minor variation in thickness causes the overall sound-absorbing curve to shift toward low frequencies, with a maximum shift of 8 Hz. The sound absorption coefficient also changes slightly, and the difference between the curves becomes more significant as the frequency increases. This indicates that the manufacturing error in the depth of the rear cavity has a minor impact on the actual measurement results. During the design of ultra-low-frequency acoustic metamaterials, changing the cavity size is the simplest and most effective method for achieving the desired properties. However, in the practical installation process, acoustic metamaterials are mostly attached between wall plates, and the depth of the rear cavity is also limited. Therefore, adjusting other structural parameters of the metamaterial by using the optimization algorithm under the maximized size is more appropriate to meet the practical requirements.

## 4. Conclusions

To improve the complex and variable noise environment in production and manufacturing industries, an acoustic metamaterial with parallel unequal cavities was designed. To ensure that the sound-absorbing capacity of the presented acoustic metamaterial met practical design requirements, the initial structural parameters were first selected, and then the metamaterial was optimized using the PSO algorithm, with the length of the embedded aperture as the optimization variable and the overall sound absorption coefficient as the optimization target. Further, the experimental and optimization results were compared. Additionally, the impact of manufacturing errors on the sound-absorbing capacity of the presented acoustic metamaterial was discussed. The main results of this study are summarized as follows.

(1) The presented acoustic metamaterial with parallel unequal cavities can absorb a low-frequency noise in subwavelength dimensions and has high strength, making it easily manufacturable using additive manufacturing technology. Furthermore, the sound-absorbing bandwidth can be expanded by arranging the unit structures with different sound absorption frequency ranges in arrays. With excellent load-bearing and sound-absorbing capacity, the material has wide application prospects in acoustic engineering and construction.
(2) The resonance frequencies of the individual cavities are designed according to the target sound absorption frequencies, and the initial structural parameters are selected with the aid of finite element analysis, which can largely reduce the burden of optimization. Then, the PSO algorithm adjusts the length of the embedded aperture to achieve a broadband, high-absorption coefficient acoustic metamaterial within the corresponding target frequency range.
(3) The consistency of the experimental results with the optimization results reflects the accuracy and reliability of the design method. According to the presented design method, the acoustic metamaterial with a thickness of only 50 mm achieves an average absorption coefficient of 0.87 from 384 Hz to 667 Hz and an average coefficient of 0.81 from 324 Hz to 503 Hz. The acoustic metamaterial with a thickness of only 70 mm achieves an average absorption coefficient of 0.83 from 265 Hz to 525 Hz. The acoustic metamaterial, with a thickness of 110 mm, achieves an average absorption coefficient of 0.82 from 156 Hz to 250 Hz.
(4) The parameter variation in different regions has varying impacts on the sound-absorbing capacity of the presented acoustic metamaterial. Among them, the change in diameter of the aperture and the small length of the embedded aperture have the most significant influence on the sound absorption coefficient, while variations in the depth of the rear cavity have a minor influence. This can guide the fabrication and processing of subsequent test samples.

Based on this study, the primary direction for further improvement involves optimizing the batch manufacturing process of acoustic metamaterials. This is attributed to the fact that additive manufacturing technology is predominantly suited for producing small quantities of samples. In contrast, the industrial-scale batch production of practical products necessitates processes such as mold manufacturing, stamping, forming, or injection molding. Above all, the proposed acoustic metamaterial with parallel unequal cavities can achieve optimal sound absorption properties in the low-frequency range with a limited total thickness for certain application scenarios, which is beneficial for improving the overall working environment and protecting the health of employees simultaneously.

## Figures and Tables

**Table 1:** Design requirements of different application scenarios.

Scenario	Installable Dimension/mm	Noise Frequency Range/Hz	Target Frequency Range/Hz	Target Sound Absorption Coefficient
1	50	400–600	350–650	≥0.8
2	50	350–500	300–550	≥0.8
3	70	300–500	250–550	≥0.8
4	110	175–250	150–300	≥0.8

**Table 2:** The structural parameters of the presented acoustic metamaterial with parallel unequal cavities.

Region	Thickness/mm	Thickness of Wall/mm	Diameter of Aperture/mm
1	50	2	2.98
2			3.65
3			3.90
4			4.14

**Table 3:** The desired initial resonance frequencies of single cavities.

Region	Serial Number	Resonance Frequency/Hz			
		Scenario 1	Scenario 2	Scenario 3	Scenario 4
1	1	450	370	360	210
	2	470	390	380	220
	3	490	410	400	230
	4	510	430	420	240
	5	530	450	440	250
	6	550	470	460	260
2	7	305	300	250	150
	8	320	305	260	155
	9	335	310	270	160
	10	350	315	280	165
3	11	370	325	295	170
	12	390	335	310	180
	13	410	345	325	190
	14	430	355	340	200
4	15	575	490	475	270
	16	600	510	500	280
	17	625	530	525	290
	18	650	550	550	300

**Table 4:** The parameters of the initial lengths of the embedded apertures.

Region	Serial Number	Initial Length of the Embedded Aperture/mm			
		Scenario 1	Scenario 2	Scenario 3	Scenario 4
1	1	6.765	12.125	7.228	15.965
	2	6.100	10.532	6.002	14.214
	3	5.401	9.025	5.098	12.658
	4	4.462	7.997	4.430	11.266
	5	3.917	6.765	3.829	10.211
	6	3.498	6.100	3.100	9.247
2	7	19.213	19.998	19.150	34.489
	8	16.990	19.213	17.348	31.964
	9	15.023	18.433	15.500	29.652
	10	13.378	17.721	14.261	27.492
3	11	13.758	19.410	14.930	30.057
	12	11.852	17.931	12.956	26.122
	13	10.303	16.678	11.472	23.001
	14	8.952	15.395	9.995	20.002
4	15	3.738	6.897	3.751	10.501
	16	3.240	6.000	2.855	9.450
	17	2.719	5.200	2.310	8.633
	18	2.289	4.543	2.000	7.496

**Table 5:** The optimized lengths of embedded apertures for the four scenarios.

Region	Serial Number	Optimized Length of Embedded Aperture/mm			
		Scenario 1	Scenario 2	Scenario 3	Scenario 4
1	1	3.41	3.49	4.92	5.78
	2	3.98	3.87	5.77	6.29
	3	4.82	4.52	6.71	7.41
	4	5.55	5.21	7.62	8.52
	5	6.39	5.88	8.50	9.39
	6	7.17	6.50	9.43	10.10
2	7	11.94	17.79	17.62	32.37
	8	12.82	18.91	18.68	33.12
	9	13.73	20.00	19.83	33.79
	10	14.55	21.15	20.95	34.51
3	11	8.31	15.18	12.78	28.21
	12	10.10	17.02	15.02	28.34
	13	11.67	18.61	17.01	32.85
	14	13.14	20.02	18.84	32.94
4	15	2.21	10.67	2.05	17.81
	16	2.89	12.51	3.19	21.16
	17	3.90	14.52	4.54	26.18
	18	5.22	15.06	5.96	26.30

## Data Availability

The original contributions presented in this study are included in the article. Further inquiries can be directed to the corresponding authors.
